# Exploring the History of Chloroplast Capture in *Arabis* Using Whole Chloroplast Genome Sequencing

**DOI:** 10.3390/ijms19020602

**Published:** 2018-02-18

**Authors:** Akira Kawabe, Hiroaki Nukii, Hazuka Y. Furihata

**Affiliations:** Faculty of Life Sciences, Kyoto Sangyo University, Kyoto, Kyoto 603-8555, Japan; g1447799@cc.kyoto-su.ac.jp (H.N.); hazuka.furi@cc.kyoto-su.ac.jp (H.Y.F.)

**Keywords:** *Arabis*, chloroplast capture, Brassicaceae

## Abstract

Chloroplast capture occurs when the chloroplast of one plant species is introgressed into another plant species. The phylogenies of nuclear and chloroplast markers from East Asian *Arabis* species are incongruent, which indicates hybrid origin and shows chloroplast capture. In the present study, the complete chloroplast genomes of *A. hirsuta*, *A. nipponica,* and *A. flagellosa* were sequenced in order to analyze their divergence and their relationships. The chloroplast genomes of *A. nipponica* and *A. flagellosa* were similar, which indicates chloroplast replacement. If hybridization causing chloroplast capture occurred once, divergence between recipient species would be lower than between donor species. However, the chloroplast genomes of species with possible hybrid origins, *A. nipponica* and *A. stelleri,* differ at similar levels to possible maternal donor species *A. flagellosa,* which suggests that multiple hybridization events have occurred in their respective histories. The mitochondrial genomes exhibited similar patterns, while *A. nipponica* and *A. flagellosa* were more similar to each other than to *A. hirsuta*. This suggests that the two organellar genomes were co-transferred during the hybridization history of the East Asian *Arabis* species.

## 1. Introduction

The genus *Arabis* includes about 70 species that are distributed throughout the northern hemisphere. The genus previously included many more species, but a large number of these were reclassified into other genera, including *Arabidopsis*, *Turritis*, and *Boechera*, *Crucihimalaya*, *Scapiarabis*, and *Sinoarabis* [1,2,3,4,5,6]. Because of their highly variable morphology and life histories, *Arabis* species have been used for ecological and evolutionary studies of morphologic and phenotypic traits [7,8,9,10,11]. The whole genome of *Arabis alpina* has been sequenced, providing genomic information for evolutionary analyses [12,13].

Molecular phylogenetic studies of *Arabis* species have been conducted to determine species classification and also correlation to morphological evolution of *Arabis* species [10,14,15]. Despite having similar morphologies, *A. hirsuta* from Europe, North America, and East Asia have been placed in different phylogenetic positions and are now considered distinct species. For example, East Asian *A. hirsuta*, which was previously classified as *A. hirsuta* var. *nipponica,* is now designated as *A. nipponica* [16]. Meanwhile, nuclear ITS sequences indicated that *A. nipponica, A. stelleri,* and *A. takeshimana* were closely related to European *A. hirsuta*. However, chloroplast *trnLF* sequences indicated that the species were closely related to East Asian *Arabis* species [14,16]. Such incongruent nuclear and organellar phylogenies have been reported from in other plant species and this is generally known as “chloroplast capture” [17,18], which is a process that involves hybridization and many successive backcrosses [17]. When chloroplast capture happens, the chloroplast genome of a species is replaced by another species’ chloroplast genome. *A. nipponica* may have originated from the hybridization of *A. hirsuta* or *A. sagittata* and East Asian *Arabis* species (similar to *A. serrata*, *A. paniculata*, and *A. flagellosa*), which act as paternal and maternal parents, respectively [14,16]. However, the evolutionary history and hybridization processes of *A. nipponica* and other East Asian *Arabis* species still need to be clarified. Because these conclusions for incongruence between nuclear and chloroplast phylogenies came from analyzing a small number of short sequences, hybridized species, the divergence level, and the classification of species are somewhat ambiguous. In the present study, the whole chloroplast genomes of three *Arabis* species were sequenced in order to analyze their divergence and evolutionary history. The whole chloroplast genome sequences also provide a basis for future marker development.

## 2. Results

### 2.1. Chloroplast Genome Structure of Arabis Species

**Figure 1:**
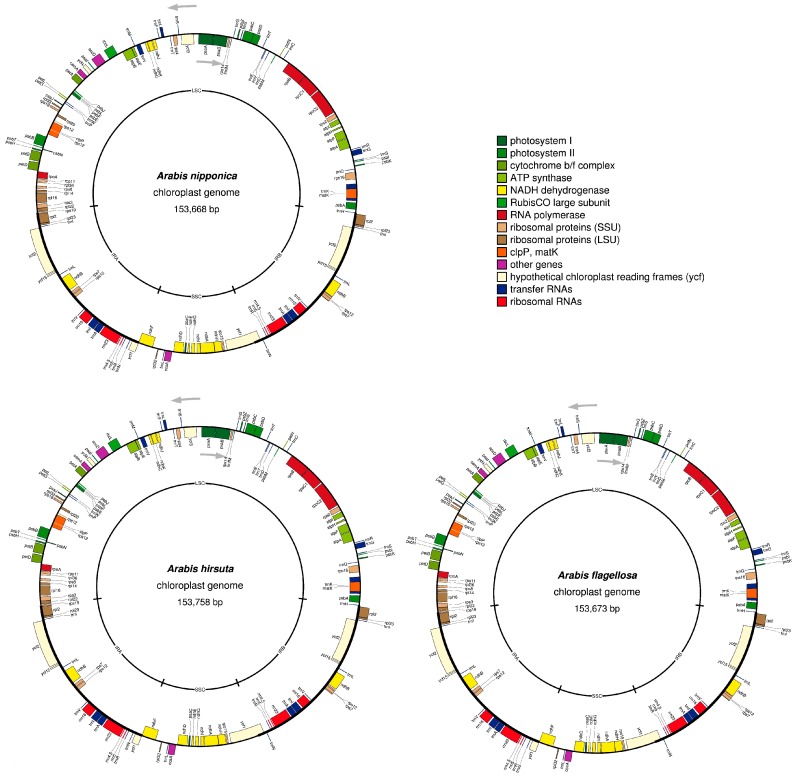
Chloroplast genome structure of *Arabis* species. Genes shown outside the map circles are transcribed clockwise, while those drawn inside are transcribed counterclockwise. Genes from different functional groups are color-coded according to the key at the top right. The positions of long single copy (LSC), short single copy (SSC), and two inverted repeat (IR: IRA and IRB) regions are shown in the inner circles.

The structures of the whole chloroplast genomes are summarized in Table 1, which also includes previously reported *Arabis* chloroplast genomes and the chloroplast genome of the closely related species *Draba nemorosa*. The chloroplast genome structure identified in the present study is shown as a circular map (see Figure 1). The complete chloroplast genomes of the *Arabis* species had total lengths of 152,866–153,758 base pairs, which included 82,338 to 82,811 base pair long single copy (LSC) regions and 17,938 to 18,156 base pair short single copy (SSC) regions, which were separated by a pair of 26,421 to 26,933 base pair inverted repeat (IR) regions. The structure and length are conserved, and are similar to other Brassicaceae species’ chloroplast genome sequences [19,20,21,22]. The complete genomes contain 86 protein-coding genes, 37 tRNA genes, and eight rRNA genes. Of these, seven protein-coding genes, seven tRNA genes, and four rRNA genes were located in the IR regions, and were therefore duplicated. The *rps16* gene became a pseudogene in *A. flagellosa*, *A. hirsuta*, and *A. nipponica* strain Midori, which was previously reported as a related species [23]. In addition, the *rps16* sequences of *D. nemorosa*, *A. stelleri*, *A. flagellosa*, *A. hirsuta*, and *A. nipponica* shared a 10 base pair deletion in the first exon, while *A. stelleri*, *A. flagellosa*, *A. hirsuta*, and *A. nipponica* shared a 1 base pair deletion in the second exon and *D. nemorosa* lacked the second exon entirely. The *rps16* sequence of *A. alpina* also lacked part of the second exon and had mutations in the start and stop codons. Therefore, different patterns of *rps16* pseudogenization were observed in *A. alpina* and the other *Arabis* species, as was previously suggested [23]. The *A. alpina* lineage had acquired independent dysfunctional mutation(s). The patterns observed for the European *A. hirsuta* revealed that the pseudogenization of *rps16* in the other *Arabis* species might not have occurred independently but, instead, occurred before the divergence of *D. nemorosa* and other Arabis species after splitting from *A. alpina*.

### 2.2. Chloroplast Genome Divergence

**Figure 2:**
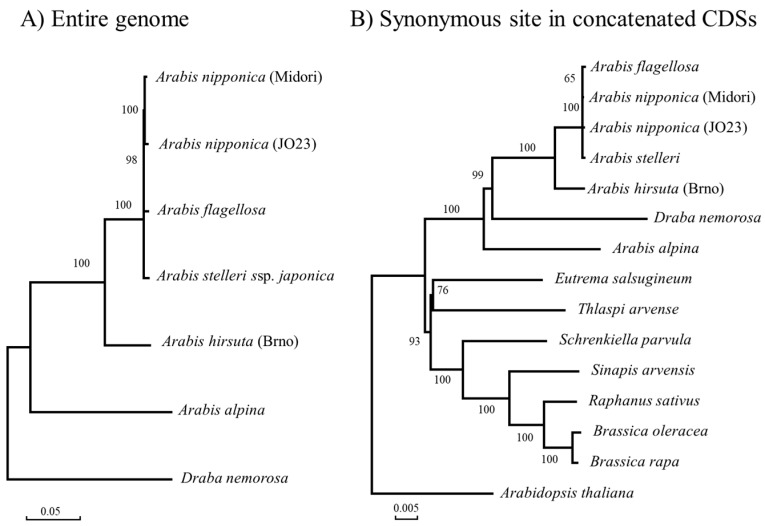
Chloroplast genome-based phylogenetic trees of *Arabis* species. The neighbor-joining trees were constructed using both (**A**) whole chloroplast genomes and (**B**) synonymous divergence from concatenated CDS. Numbers beside the nodes indicate bootstrap probabilities (%). Scale bars are shown at the bottom left of each tree.

Phylogenetic trees were generated by using whole chloroplast genome sequences and concatenated coding sequence (CDS) regions (see Figure 2). The inclusion of other Brassicaceae members revealed that *D. nemorosa* should be placed within *Arabis*, as previously reported [24]. In both trees, the two *A. nipponica* strains were grouped with *A. flagellosa* and *A. stelleri*. Although several nodes were supported by high bootstrap probabilities, the nearly identical sequences of the four East Asian *Arabis* species made them indistinguishable.

**Figure 3:**
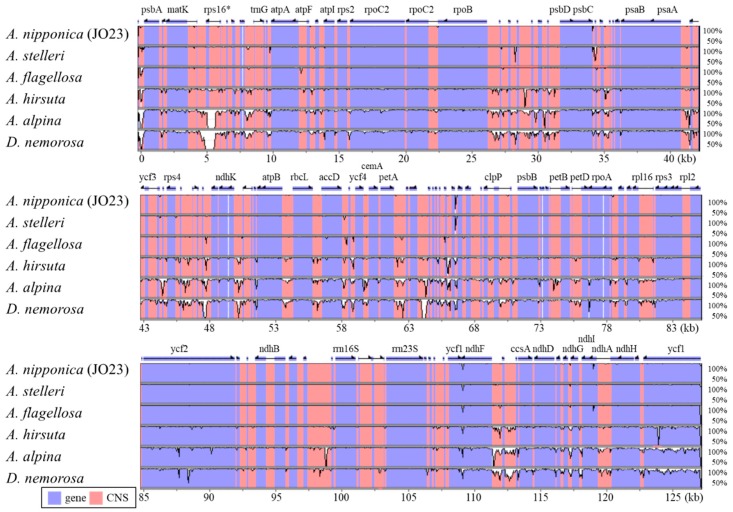
Alignment of the seven chloroplast genomes. VISTA-based identity plots of chloroplast genomes from six *Arabis* species and *Draba nemorosa* are compared to *A. nipponica* strain Midori. Arrows above the alignment indicate genes and their orientation. The names of genes ≥500 bp in length are also shown. A 70% identity cut-off was used for making the plots, and the Y-axis represents percent identity (50–100%), while the X-axis represents the location in the chloroplast genome. The blue and pink regions indicate genes and conserved noncoding sequences, respectively.

The divergence among the *Arabis* chloroplast genomes was shown using a VISTA plot (see Figure 3) and this was summarized in Table 2. The genome sequences of the two Japanese *A. nipponica* strains differed by only 55 nucleotide substitutions (0.036% per site), while those of *A. hirsuta* and *A. nipponica* differed by about 3500 sites (2.4% per site). The chloroplast genomes of *A. nipponica* and the other two East Asian *Arabis* species were also very similar (~100 nucleotide differences, <0.1% per site). Additionally, the 35 CDS regions, 29 tRNA genes, and four rRNA genes of the four East Asian *Arabis* species were identical, with three, 27, and four, respectively, also found to be identical in *A. hirsuta*. The levels of divergence between the East Asian *Arabis* species were similar to previously reported levels of variation within the local *A. alpina* population, in which 130 SNPs were identified among 24 individuals (Waterson’s *θ* = 0.02%) [25]. If the hybridization event had facilitated chloroplast capture, the divergence between the *A. stelleri* and *A. nipponica* chloroplast genomes should have been less than their divergence from *A. flagellosa*. However, the divergence between the potential hybrid-origin species (*A. stelleri* and *A. nipponica*: 0.068 to 0.085) was similar to their divergence from *A. flagellosa* (0.056 to 0.086). Although the level of divergence was too low to make reliable comparisons, it is possible that *A. stelleri* and *A. nipponica* originated from independent hybridization events or the introgression process may still be ongoing.

### 2.3. Distribution of Simple Sequence Repeats in the Chloroplast Genomes

Because the extremely low divergence among the East Asian *Arabis* species made it difficult to resolve their evolutionary relationships, other highly variable markers were needed. Therefore, simple sequence repeat (SSR) regions throughout the chloroplast genome were assessed for their ability to provide high-resolution species definition. A total of 74 mono-nucleotide, 22 di-nucleotide, and two tri-nucleotide repeat regions of ≥10 base pairs in length were identified (see Table 3). However, these repeat regions were still unable to completely resolve the relationships of the East Asian *Arabis* species. Fifty of the 98 SSRs exhibited no variation among the East Asian *Arabis* species, while only 29 SSRs exhibited species-specific variation, including nine in *A. flagellosa*, 15 in *A. stelleri*, four in *A. nipponica* strain JO23, and one in *A. nipponica* strain Midori. Five of the SSRs were shared by the two *A. nipponica* strains, which suggests that they were also species-specific. Although the two *A. nipponica* strains were similar to each other, *A. flagellosa*, *A. stelleri*, and *A. nipponica* differ to a similar degree in terms of of variable SSRs, which suggests that the occurrence of chloroplast capture would be independent or still ongoing. This was suggested by the patterns of nucleotide substitutions.

### 2.4. Mitochondrial Genome Analysis

Chloroplast capture could have originated from hybridization events that also affected other cytoplasmic genomes. Due to this, variation in the mitochondrial genome sequences was analyzed. Mapping next-generation sequencing (NGS) reads to the *Eruca vesicaria* mitochondrial genome revealed that 29 sites with five or more mapped reads varied among the *A. nipponica* strain Midori, *A. flagellosa*, and *A. hirsuta* (see Table 4). Twenty-eight of the sites were conserved among *A. nipponica* and *A. flagellosa.* One site was specific to *A. nipponica* and provided 100% support for the relationship between *A. nipponica* and *A. flagellosa*. Even though reliability decreased, 123 of 125 sites with two or more reads (98.4%) also supported the similarity of the *A. nipponica* and *A. flagellosa* mitochondrial genomes. These findings suggest that the hybridization history of the species affects both the chloroplast and the mitochondrial genomes similarly.

## 3. Discussion

Chloroplast capture results in the incongruence of chloroplast and nuclear phylogenies, which has been reported in many plant taxa and is considered common among plants [17,18,26,27,28,29,30,31,32,33,34,35,36,37]. Furthermore, it is possible that the introgression of chloroplast genomes occurs more frequently than that of nuclear genomes as a result of uniparental inheritance, lack of recombination, and low selective constraint [38,39,40]. Chloroplast capture could occur by using several factors including sampling error, convergence, evolutionary rate heterogeneity, wrong lineage sorting, and hybridization/introgression [17]. Introgression-induced chloroplast capture occurred through hybridization between distant but compatible species, which was followed by backcrossing with pollen donor species [41,42].

East Asian *Arabis* species have previously been reported to show evidence of chloroplast capture [14,16]. More specifically, detailed phylogenetic analyses of nuclear and chloroplast marker genes has suggested that *A. nipponica, A. stelleri,* and *A. takeshimana* originated from the hybridization of *A. hirsuta* (or *A. sagittata*) and East Asian *Arabis* species (close to *A. serrata*, *A. paniculata*, and *A. flagellosa*), which act as paternal and maternal parents, respectively [14,16]. In the present study, comparing the whole chloroplast genomes of four plants from three East Asian *Arabis* species (two *A. nipponica*, one each of *A. stelleri*, and *A. flagellosa*) revealed genome-wide similarities that indicated chloroplast capture by *A. nipponica* and *A. stelleri*. The study also compared the species’ partial mitochondrial genomes, which indicated a closer relationship between *A. nipponica* and *A. flagellosa* than between the former and European *A. hirsuta*. This suggested that *A. nipponica* also has a history of mitochondrial capture. This is not surprising, because hybridization and backcrossing could have similar effects on both organellar genomes. Also, cyto-nuclear incompatibility caused by a mitochondrial genome could lead cytoplasmic replacement to exhibit chloroplast capture [17,41,42]. The pattern of variation in the mitochondrial genomes suggested that both the chloroplast and mitochondrial genomes were co-transmitted during the evolutionary history of East Asian *Arabis* species. Future research should focus on the process of chloroplast (organellar) capture. Simple backcrossing could show the mechanisms of cytoplasm replacement and could produce results in as few as a hundred generations under certain conditions [42]. In the present study, the divergence between the genomes of hybrid-origin species and putative pollen-donor species was similar to the divergence observed within species, which suggests that the hybridization event was relatively recent. Nuclear genome markers are needed to estimate the proportion of parental genome fragments in the current nuclear genome of *A. nipponica*.

## 4. Materials and Methods

### 4.1. Plant Materials

*Arabis nipponica* (*A. hirsuta* var. *nipponica*, sampled from Midori, Gifu Prefecture, Japan), *A. flagellosa* (sampled from Kifune, Kyoto Prefecture, Japan), and *A. hirsuta* (strain Brno from Ulm Botanical Garden, Germany) were used in the present study.

### 4.2. DNA Isolation, NGS Sequencing, and Genome Assembly

Chloroplasts were isolated from *A. hirsuta* and *A. nipponica* as described in Okegawa and Motohashi [43]. DNA was isolated from the chloroplasts using the DNeasy Plant Mini Kit (Qiagen, Valencia, CA, USA), while the total DNA was isolated from leaves of *A. flagellosa*. NGS libraries were constructed using the Nextera DNA Sample Preparation Kit (Illumina, San Diego, CA, USA) and sequenced as single-ended reads using the NextSeq500 platform (Illumina). About 2 Gb (1.4 Gb, 12 M clean reads) of sequences were obtained for *A. flagellosa* (43 Mb mapped reads, 282.69× coverage). Additionally, 400 Mb (300 Mb, 2.5 M clean reads) were obtained for both *A. hirsuta* (64 Mb mapped reads, 417.17× coverage) and *A. nipponica* (72 Mb mapped reads, 455.87× coverage). The generated reads were assembled using velvet 1.2.10 [44] and assembled into complete chloroplast genomes by mapping to previously published whole chloroplast genome sequences. Sequence gaps were resolved using Sanger sequencing. Genes were annotated using DOGMA [45] and BLAST. The newly constructed chloroplast genomes were deposited in the DDBJ database under the accession numbers LC361349-51. Finally, the circular chloroplast genome maps were drawn using OGDRAW [46].

### 4.3. Molecular Evolutionary Analyses

The whole chloroplast genome sequences of *A. nipponica* (strain JO23: AP009369), *A. stelleri* (KY126841) [23], *A. alpina* (HF934132) [25], and *D. nemorosa* (strain JO21: AP009373) in the GenBank were also used. Whole chloroplast sequences were aligned in order to construct neighbor-joining trees with Jukes and Cantor distances. The sequences of 77 known functional genes were linked in a series after excluding initiation and stop codons and were then used for phylogenetic analyses along with sequences from the related clade species *Brassica oleracea* (KR233156) [47], *B. rapa* (DQ231548), *Eutrema salsugineum* (KR584659) [48], *Raphanus sativus* (KJ716483) [49], *Scherenkiella parvula* (KT222186) [48], *Sinapis arvensis* (KU050690), and *Thlaspi arvense* (KX886351) [21] using *A. thaliana* (AP000423) [50] as an outgroup. The synonymous divergence of the concatenated CDS was estimated using the Nei and Gojobori method. All phylogenetic analyses were performed using MEGA 7.0 [51]. Levels of divergence throughout the chloroplast genome were visualized using mVISTA [52] with Shuffle-LAGAN alignment [53].

### 4.4. Mapping NGS Reads to Mitochondrial Genome Sequences

Because the chloroplast isolation method used in the present study did not completely exclude mitochondria, about 1% of the sequence reads were derived from mitochondrial genomes. Although this proportion is too low to be useful for assembling whole mitochondrial genomes, the reads were nevertheless mapped to the mitochondrial genome of *Eruca vesicaria* (KF442616) [54] in order to measure mitochondrial genome divergence. Regions with at least five mapped reads were used for the analysis.

## Figures and Tables

**Table 1:** Summary of chloroplast genome structure in *Arabis* species.

Species	Strain	Nucleotide Length (bp)				GC Contents (%)				NCBI #	Reference
		Entire	LSC	SSC	IR	Entire	LSC	SSC	IR		
*Draba nemorosa*	JO21	153289	82457	18126	26353	36.47	34.27	29.3	42.39	AP009373 (NC009272)	
*Arabis alpina*		152866	82338	17938	26933	36.45	34.21	29.31	42.39	HF934132 (NC023367)	[25]
*Arabis hirsuta*	Brno	153758	82710	18156	26446	36.4	34.15	29.16	42.41	LC361350	this study
*Arabis flagellosa*	Kifune	153673	82775	18052	26423	36.4	34.13	29.22	42.41	LC361351	this study
*Arabis stelleri*		153683	82807	18030	26423	36.39	34.11	29.22	42.42	KY126841	[23]
*Arabis nipponica*	JO23	153689	82811	18036	26421	36.4	34.1	29.31	42.42	AP009369 (NC009268)	
*Arabis nipponica*	Midori	153668	82772	18052	26422	36.39	34.1	29.24	42.42	LC361349	this study

**Table 2:** Divergence between species.

Compared Species			# of Differences	Divergence (%: Ks with JC Correction)
*Draba nemorosa*	vs.	*Arabis alpina*	4475	2.976
*Draba nemorosa*	vs.	*Arabis hirsuta*	4219	2.801
*Draba nemorosa*	vs.	*Arabis flagellosa*	4262	2.765
*Draba nemorosa*	vs.	*Arabis stelleri*	4171	2.771
*Draba nemorosa*	vs.	*Arabis nipponica* (JO23)	4150	2.757
*Draba nemorosa*	vs.	*Arabis nipponica* (Midori)	4131	2.745
*Arabis alpina*	vs.	*Arabis hirsuta*	3566	2.366
*Arabis alpina*	vs.	*Arabis flagellosa*	3571	2.371
*Arabis alpina*	vs.	*Arabis stelleri*	3565	2.366
*Arabis alpina*	vs.	*Arabis nipponica* (JO23)	3564	2.366
*Arabis alpina*	vs.	*Arabis nipponica* (Midori)	3547	2.355
*Arabis hirsuta*	vs.	*Arabis flagellosa*	1245	0.815
*Arabis hirsuta*	vs.	*Arabis stelleri*	1253	0.82
*Arabis hirsuta*	vs.	*Arabis nipponica* (JO23)	1234	0.808
*Arabis hirsuta*	vs.	*Arabis nipponica* (Midori)	1214	0.795
*Arabis flagellosa*	vs.	*Arabis stelleri*	132	0.086
*Arabis flagellosa*	vs.	*Arabis nipponica* (JO23)	111	0.072
*Arabis flagellosa*	vs.	*Arabis nipponica* (Midori)	86	0.056
*Arabis stelleri*	vs.	*Arabis nipponica* (JO23)	130	0.085
*Arabis stelleri*	vs.	*Arabis nipponica* (Midori)	104	0.068
*Arabis nipponica* (JO23)	vs.	*Arabis nipponica* (Midori)	55	0.036

**Table 3:** Simple sequence repeats (SSRs) in *Arabis* chloroplast genome.

Position in *A. nipponica* (Midori) Genome		UNIT		*A. nipponica*		*A. stelleri*	*A. flagellosa*	*A. hirsuta*	*A. alpina*
from	to			Midori	JO23				
287	318	AT		16	15	15	12	13 with 2 mutations	29 bp with several mutations
1922	1932	A		11	11	9	12	11	9
3929	3938	T		10	9	10	9	7	7
4258	4270	T		13	18	18	17	13	13
7713	7727	T		15	15	15	15	12	11
7729	7738	A		10	10	10	9	10	10
8203	8216	TA		7	6	7	7	6	6
8273	8282	TA		5	5	5	5	5	6
8289	8302	AT		7	7	6	7	8	6
8321	8330	TA		5	5	4	5	5	deletion
9677	9690	T		14	14	T4GT10	15	14	14
9982	9991	TA		5	5	5	5	5	5
11,660	11,669	A		10	10	9	10	10	7
12,406	12,414	T		9	9	10	10	T3AT6	T3AT6
13,010	13,018	T		9	9	10	9	T7AT2	T10AT2
13,810	13,821	ATT		4	4	4	4	4	ATTATATTCTT
14,101	14,110	A		10	10	14	10	12	10
18,027	18,037	T	CDS	11	11	11	11	11	11
19,398	19,408	TA		5	5	5	5	5	5
22,549	22,558	T		10	10	11	10	9	15
25,777	25,786	T	CDS	10	10	10	10	10	10
27,601	27,611	G		11	11	11	15	12	9
28,808	28,817	T		10	10	9	10	10	T5CT3G2
30,293	30,310	A		18	17	17	18	12	A4CA5
30,737	30,751	T		15	15	14	15	15	5
30,830	30,839	A		10	9	11	10	8	6
30,918	30,929	TA		6	6	6	6	6	4
31,260	31,269	AT		5	5	5	5	5	3
35,309	35,316	G		8	11	10	11	7	10
35,516	35,525	AT		5	5	5	5	5	5
35,538	35,555	AT		9	9	9	9	3	deletion
41,508	41,522	T		15	13	11	13	18nt	101nt
41,768	41,778	A		11	12	12	11	A12GA4	11
43,656	43,665	A		10	10	10	11	A8TA2	A9TA2TA2
43,887	43,895	T		9	15	T4AT4AT4	T4AT4	4	4
45,038	45,046	T		9	9	9	10	8	7
45,771	45,788	T		18	18	18	18	13	9
46,034	46,057	A		24	24	24	24	16	11
46,116	46,133	AT		9	9	9	8	9	7
46,135	46,144	TA		5	5	5	5	5	3
46,782	46,791	T		10	11	10	11	14	10
47,368	47,378	A		11	11	12	12	10	13
47,586	47,595	T		10	10	10	10	13	TCT8
47,624	47,633	A		10	10	11	11	8	7
49,061	49,070	T		10	10	11	10	8	T3AT10
49,631	49,640	T		10	10	10	10	8	8
50,329	50,340	A		12	12	12	12	11	11
51,202	51,211	TA		5	5	5	5	19nt	deletion
51,215	51,230	T		16	17	17	16	13	13
53,088	53,097	T	CDS	10	10	10	10	10	12
53,592	53,601	C		10	11	9	12	9	9
55,477	55,490	T		14	14	14	14	complement A11	complement A13
55,891	55,906	T		16	16	16	16	13	15
56,476	56,485	T		10	10	10	10	10	A2T8
58,301	58,310	T		10	10	10	10	10	6
59,338	59,348	T		11	10	9	11	11	4
61,731	61,739	C		9	13	9	12	8	C3AC3
62,108	62,117	TA		5	5	5	5	6	4
62,161	62,182	T		22	22	22	31	2nt shorter	2nt shorter
62,202	62,210	A		9	10	9	9	A5TA3	A5TA3
63,523	63,538	T		16	15	15	T5GT10	16	7
64,629	64,639	T		11	11	11	11	11	T6GT3G
65,636	65,645	C		10	13	11	13	8	C2TCTGC7
66,253	66,262	AT		5	5	5	5	4	7
66,851	66,864	A		14	14	14	19	17	12
68,965	68,977	T		13	13	13	13	11	11
69,965	69,975	T		11	11	12	11	11	8
75,328	75,340	A		13	14	14	13	19	14
76,614	76,626	T		13	13	13	13	13	13
78,154	78,162	TTG		3	5	3	3	4	2
80,484	80,493	A		10	11	10	10	10	9
81,019	81,035	T		17	17	17	17	17	17
81,178	81,191	T		14	14	14	14	18	8
82,568	82,578	A		11	10	9	10	9	10
83,489	83,498	TA		5	5	5	5	5	4
93,127	93,136	TA		5	5	5	5	5	4
97,975	97,984	A		10	10	10	10	12	9
98,781	98,791	T		11	11	11	11	10	14
107,287	107,295	AT		5	5	5	5	5	7
107,313	107,324	T		12	11	13	13	T2(AT)4T7	14
111,481	111,490	TA		5	5	5	5	TA2TGTA	4
111,589	111,598	AT		5	5	5	5	5	10
111,665	111,672	T		8	8	10	8	7	10
111,801	111,810	A		A7CA2	A7CA2	10	A7CA2	A7CA2	A7TAC
112,472	112,481	A		10	10	10	10	11	10
116,836	116,845	T		10	9	10	11	T7AT3	10
123,173	123,184	T		12	12	12	12	12	12
123,285	123,383	T		10	10	10	10	10	10
123,884	123,893	T		10	10	10	10	10	10
123,975	123,987	A		13	13	13	13	13	13
124,356	124,365	TA		5	5	5	5	5	5
124,874	124,886	T		13	13	13	13	13	13
125,029	125,041	A		13	13	13	13	13	13
126,052	125,385	T		15	15	15	15	15	17
126,087	126,097	T		11	11	11	11	11	11
126,117	126,128	A		12	12	12	12	12	12
126,952	126,962	T		11	11	11	11	T8CT2	T8CT2
127,241	127,252	A		12	12	12	12	6	6

**Table 4:** Nucleotide variation in the mitochondrial genome of *Arabis* species.

		Number of Mapped Reads			
		5 and More	4 and More	3 and More	2 and More
Number of variable sites	Total	29	46	74	129
Specific to	*A. nipponica*	1	1	4	12
	*A. flagellosa*	0	0	0	3
	*A. hirsuta*	14	25	35	62
Shared with	*A. flagellosa* and *A. nipponica*	14	19	31	46
	*A. nipponica* and *A. hirsuta*	0	0	1	1
	*A. flagellosa* and *A. hirsuta*	0	0	1	1
	other type	0	1	2	4
